# Endomembrane proteomics reveals putative enzymes involved in cell wall metabolism in wheat grain outer layers

**DOI:** 10.1093/jxb/erv075

**Published:** 2015-03-13

**Authors:** Anne-Laure Chateigner-Boutin, Muhtadi Suliman, Brigitte Bouchet, Camille Alvarado, Virginie Lollier, Hélène Rogniaux, Fabienne Guillon, Colette Larré

**Affiliations:** INRA, UR1268 Biopolymères, Interactions Assemblages, F-44316 Nantes, France

**Keywords:** Carbohydrate active enzymes, cell wall, Golgi apparatus, proteomics, subcellular fraction, wheat grain outer layers.

## Abstract

Cereal grain outer layers fulfil essential functions for the developing seed such as supplying energy and providing protection. In the food industry, the grain outer layers called ‘the bran’ is valuable since it is rich in dietary fibre and other beneficial nutriments. The outer layers comprise several tissues with a high content in cell wall material. The cell wall composition of the grain peripheral tissues was investigated with specific probes at a stage of active cell wall synthesis. Considerable wall diversity between cell types was revealed. To identify the cellular machinery involved in cell wall synthesis, a subcellular proteomic approach was used targeting the Golgi apparatus where most cell wall polysaccharides are synthesized. The tissues were dissected into outer pericarp and intermediate layers where 822 and 1304 proteins were identified respectively. Many carbohydrate-active enzymes were revealed: some in the two peripheral grain fractions, others only in one tissue. Several protein families specific to one fraction and with characterized homologs in other species might be related to the specific detection of a polysaccharide in a particular cell layer. This report provides new information on grain cell walls and its biosynthesis in the valuable outer tissues, which are poorly studied so far. A better understanding of the mechanisms controlling cell wall composition could help to improve several quality traits of cereal products (e.g. dietary fibre content, biomass conversion to biofuel).

## Highlight

Wheat grain outer layers, decisive for development, protection and end-uses, comprise several specialized layers. Cell wall heterogeneity is highlighted and correlated to probable differences in composition of wall machineries.

## Introduction

Cell walls are complex structures that envelop all plant cells. The main components of plant cell walls are polysaccharides, proteins and, in some specialized cells, non-carbohydrate substances (e.g. lignins). Considerable variations have been observed in cell wall composition between species and within a given plant species between tissues and developmental stages. Cell wall composition and architecture affect its properties *in planta* and also in crop products (e.g. degradation potential of plant byproducts for biofuel production, dietary fibre quality of cereal products).

The wheat grain is a caryopsis composed of an embryo which develops into the future wheat plant, the endosperm where metabolites are stored until remobilization following germination to sustain the seedling growth, and outer layers which mainly fulfil a nutritive function in grain at the early stage of development. In the dry grain, the outer layers have a protective role and with the endosperm-derived aleurone layer they constitute the bran. These outer layers are composed of different tissues: the pericarp, which is divided into the outer pericarp and inner pericarp, the testa or seed coat and the nucellar epidermis (Gassner 1973; Xiong *et al.*, 2013).

The composition of cell walls in wheat grain outer layers has been revealed in several studies using neutral sugar and phenolics analyses, which have revealed that cell wall composition varies considerably between the different layers (Antoine *et al.*, 2003; Beaugrand *et al.*, 2004; Parker *et al.*, 2005; Barron *et al.*, 2007).

The metabolism of cell wall polysaccharides requires the intervention of numerous enzymes. Enzymes acting on carbohydrates are listed and classified on the basis of their sequence similarities and activities in the CAZy database (http://www.CAZy.org/) (Cantarel *et al.*, 2009). Cell wall polysaccharides are synthesized in two major subcellular locations. While cellulose and callose are produced at the plasma membrane surface (Mueller and Brown, 1980; Cai *et al.*, 2011), pectin and hemicellulose are synthesized in the Golgi apparatus to then be transported to the wall (Northcote and Pickett-Heaps, 1966).

The glycosyltransferase (GT) superfamily contains enzymes that are involved in cell wall polysaccharide synthesis and several have been localized in the Golgi apparatus (Anders *et al.*, 2012; Lee *et al.*, 2014). In addition to GTs, other families of proteins are involved in cell wall polysaccharide maturation or remodelling, and in cell wall degradation. These proteins are glycosylhydrolases (GHs), esterases, and other transferases. They act on cell wall polysaccharides either in the Golgi apparatus (e.g. pectin methyl esterase PME), or in the cell wall (e.g. xyloglucan endotransglycosylase/hydrolase XET/XTH) (Franková and Fry, 2013).

To gain access to the cellular machinery involved in the synthesis of the cell walls in the wheat grain, we conducted a proteomic approach on wheat outer layers from developing grain harvested at a stage where cell wall polysaccharides are accumulating. Because many GTs are membrane proteins characterized by issues such as low abundance and poor solubility, fractions enriched in Golgi membranes were isolated. Proteins were extracted from these outer layers, analysed by mass spectrometry and more than a thousand proteins were identified. Among these proteins some are homologs to CAZy proteins known to be involved in cell wall metabolism and others may represent interesting new candidates to investigate. In this paper we discuss cell wall-related enzymes in relation to polymers highlighted by microscopy experiments or described in the literature.

## Materials and methods

### Plant materials

The plants *Triticum aestivum* L. cv. Recital were grown in pots in a greenhouse under conditions of natural day length (UMR Amélioration des Plantes et Biotechnologies Végétales, INRA-Rennes, France). To define developmental stages, individual ears were tagged at flowering. Seed development was calculated on the basis of cumulative temperature in Celsius degrees days (°D) after flowering. Grains were harvested at 250–275°D and manually dissected to separate outer pericarp, intermediate layers and endosperm. The dissected tissues were stored in buffer A (250mM sucrose, 10mM HEPES-NaOH, pH 7.4, 1mM EDTA, 1mM DTT and anti-protease cocktail) (Roche) on ice before further treatment. For microscopy studies, wheat grains were harvested at 250°D.

### Microscopy

#### High-pressure freezing and substitution

Cross sections (150 µm) made with a vibratome (HM 650V, Microm) were punched with a 1.5 mm-diameter biopsy punch (Miltex). Punches were transferred to 200 μm-deep flat carriers (Leica Microsystems) filled with hexadecene-1 (Sigma), cryo-immobilized at 2000 bar pressure using an EM-PACT2 (Leica Microsystems) and rapidly transferred to liquid nitrogen. Freeze substitution was carried out using an EM-AFS2 system (Leica Microsystems) in anhydrous acetone in 0.2% uranyl acetate (Electron Microscopy Sciences) and 0.2% glutaraldehyde (Agar Scientific) at −90°C for 100h, followed by a gradual temperature increase to −50°C. The samples were washed with pure ethanol for 48h and infiltrated with Lowicryl HM20 (Electron Microscopy Sciences) according to the following schedule: 20% resin in ethanol (2h), 40% resin (3h), 60% resin (4h), 80% resin (16h) and 100% resin (48h). Polymerization was achieved by UV light illumination at −50°C for 72h.

#### Brightfield imaging

Cross sections (20 µm) were made with a cryotome (HM 500 OM, Microm). Semi-thin embedded sections (1 µm, ultracut UC7, Leica Microsystems) stained with Toluidine Blue O (1% in 0.02% Na_2_CO_3_) for 1min and cryo-sections were observed using brightfield microscopy (DMRD, Leica Microsystems).

#### Immunolabelling

Ultra-thin sections (80nm) were cut from embedded samples and collected on nickel grids. Blocking and immunolabelling were performed as described in Chateigner-Boutin *et al.* (2014). The antibodies, probe and dilutions are described in Supplementary Table S1. Control experiments were performed omitting the primary antibodies.

### Preparation of microsomal fractions

Intermediate layers and outer pericarps were dissected from ~1000 grains and their microsomal fractions prepared according to the protocol described in Suliman *et al.* (2013) with slight modifications for outer pericarps. Outer pericarps were more difficult to homogenize; therefore to obtain a lysate three pulses of 10 s at 9000rpm using a polytron (Kinematica AG, Dispersing and Mixing Technology) were required. The lysates from intermediate layers and pericarps were then centrifuged twice for 5min at 2200 *g* and 15min at 3000 *g*. Supernatants were then loaded on the top of an SW41 centrifuge tube that contained 6ml of 18% iodixanol prepared from Optiprep solution (Sigma-Aldrich) and centrifuged at 100 000 *g* for 2h at 4°C (Beckman Coulter SW41 rotor). The interfacial fraction was collected as the microsomal fraction.

### Western blot analysis

Aliquots of proteins were separated by SDS-PAGE on 12% acrylamide gels in reducing conditions and electroblotted on nitrocellulose membranes for immunodetection according to Suliman *et al.* (2013). The specificity of the primary antibodies, anti-binding immunoglobulin protein 2 (BiP2) and anti-reversibly glycosylated protein 1 (RGP1), used for incubation as well as their dilution, are described in Supplementary Table S1. The secondary antibodies, consisting of peroxidase-conjugated anti-rabbit IgG, were used at 1:100 000 dilution, for 2h at room temperature. After washing, nitrocellulose membranes were incubated in a chemiluminescent substrate (Super Signal West Dura Extended Duration Substrate, Pierce) according to the manufacturer’s instructions. Luminescence was detected using a camera (Luminescent Image Analyzer LAS 3000; Fuji Film).

### Generation of Golgi-enriched fractions from intermediate layers

Four millilitres of the microsomal fraction from intermediate layers were further fractionated using iodixanol density gradient. Fifteen fractions were collected and tested by immunoblotting for RGP and BiP2 content.

### Protein extraction

Three fractions that exhibited a relative high Golgi apparatus content and the microsomal fractions were diluted with cold deionized water to a final volume of 22ml and ultracentrifuged at 100 000 *g* for 2h. The proteins were extracted from the Golgi-enriched endomembrane pellet with 2% SDS according to the workflow described in Supplementary Fig. S1. In the case of the outer pericarp microsomal fraction, proteins were sequentially extracted (water, 150mM Na_2_CO3 then 2% SDS) from the pelleted endomembranes generating three protein extracts (Supplementary Fig. S1). These extraction steps aimed to decrease the sample complexity.

### Analysis of peptides by mass spectrometry

Extracted proteins were briefly separated on 12% SDS-PAGE. Each lane was then systematically cut into 15–20 slices. In-gel digestion was performed after reduction and alkylation according to Larré et al. (2010).

Nanoscale LC-MS/MS analyses of the digested proteins were performed using an Ultimate 3000 RSLC system (Dionex) coupled with an LTQ-Orbitrap VELOS mass spectrometer (Thermo Fisher). Chromatographic separation was conducted on a reverse-phase capillary column (Acclaim Pepmap C18 2 µm 100A, 75 µm i.d.×15cm length; Dionex) at a flow rate of 300 nl min^-1^ as described in Suliman *et al.* (2013).

Mass data acquisitions were performed using Xcalibur 2.1 software. Full MS scans were acquired at high resolution (FWMH 30 000) in the Orbitrap analyser [mass-to-charge ratio (m/z)=400–2000], while collision-induced dissociation (CID) spectra were recorded on the five most intense ions in the linear LTQ traps.

### Database search and interpretation

Raw data collected during LC-MS/MS analyses were processed into mgf format files and further searched against databanks using X!Tandem Spectrum Modeler. Protein identification was achieved by querying mass data (MS and MS/MS spectra) against UniProt Knowledgebase (http://www.uniprot.org/) restricted to Poaceae (version 2014-12) and a contaminant database including keratins and trypsin.

A database search was performed with XTandem Sledgehammer 2013.09.01.1 (http://www.thegpm.org/TANDEM/) via the X!tandem pipeline available at http://pappso.inra.fr/bioinfo/xtandempipeline/. Enzymatic cleavage was declared as a trypsin digestion with one possible miscleavage event. Fixed modifications of cysteine residues by iodoacetamide as well as oxidized methionins were considered. Precursor mass and fragment mass tolerance were set at 5 ppm and 0.8Da, respectively. Identified proteins were filtered according to the following specifications: at least two peptides in the same band with an E value below 0.01 and a protein E value below 10^–4^. Two sets of data were generated, one for the outer pericarp obtained by combining the results from the three sequential protein extracts, and one for the intermediate layers obtained by combining the results from three enriched Golgi fractions. To take redundancy into account, in each set, proteins with at least one peptide in common were grouped. The number of identified proteins given in this report corresponds to the number of protein groups. In some groups, sub-groups of proteins were defined. The proteins in these sub-groups shared some peptides with the other proteins of the group but were distinguished by at least one specific peptide.

Proteins (groups) potentially involved in lignin and cutin metabolism were identified through their annotation. Identification of enzymes acting on carbohydrates was performed by blasting one protein per sub-group against the entire non-redundant sequences of the CAZy database using protein domains (Pfam) association rules in CAZymes Analysis Toolkit (Park *et al.*, 2010). CAZy hits (e-value<0.01) were gathered with their annotation. The annotation of proteins belonging to families implicated in cell wall metabolism was subsequently checked using tBLASTN (Altschul *et al.*, 1997) against NCBI nr restricted to Viridiplantae. When the proposed activity had no association with cell wall metabolism, the protein was not retained. For the enzymes of interest, all the protein hits were compared between pericarp and intermediate layers.

## Results

### The wheat grain outer layers at 250°D contain several cell types which actively synthesize cell wall polymers

**Fig. 1.**
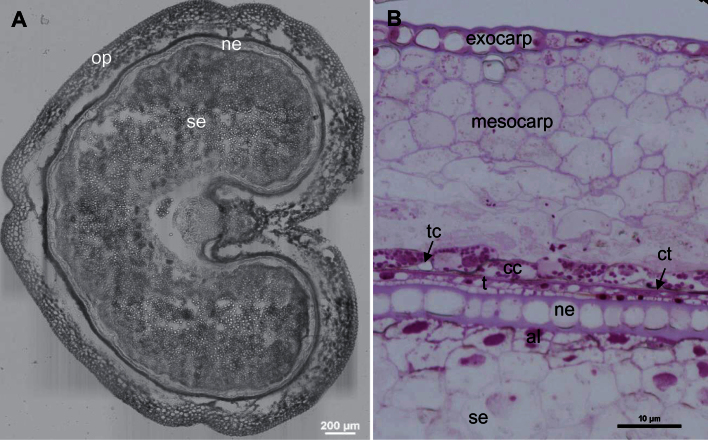
Wheat grain structure at 250°D. Brightfield image of a grain cross section (A) without staining and (B) with staining with Toluidine blue. Op, outer pericarp; ne, nucellar epidermis; se, starchy endosperm; cc, cross cells; tc, tube cell; t, testa; ct, cuticle; al, aleurone layer. This figure is available in colour at *JXB* online.

During development, the wheat grain undergoes considerable changes. Fig. 1A depicts the structure of the wheat caryopsis at 250°D after anthesis. At this stage, the endosperm comprises the starchy endosperm and the aleurone layer. The outer pericarp comprises the exocarp (the outermost pericarp layer or epiderm) and mesocarp (the middle pericarp), which appears disorganized at its base (Fig. 1A, B). The inner pericarp encompasses a layer of cross cells and and on their inner side longitudinal cells named tube cells. The testa contains two cell layers and a thick cuticle is deposited on its outer surface (Fig. 1B). The nucellar epidermis consists of a thick-walled cell layer (Fig. 1B) and a thin cuticle not seen in Fig. 1 is also deposited on its outer surface.

Wheat grains were manually dissected to harvest three fractions: outer pericarp, intermediate layers and endosperm (Supplementary Fig. S2). At 250°D these tissues are easily dissected, the outer pericarp peels off due to the degradation of the mesocarp (Xiong *et al.*, 2013). The intermediate layers are easily detached from the endosperm. Brightfield micrograph reveals that they contains several cell layers corresponding to the nucellar epidermis, the testa, tube cells and cross cells (Supplementary Fig. S2).

**Fig. 2.**
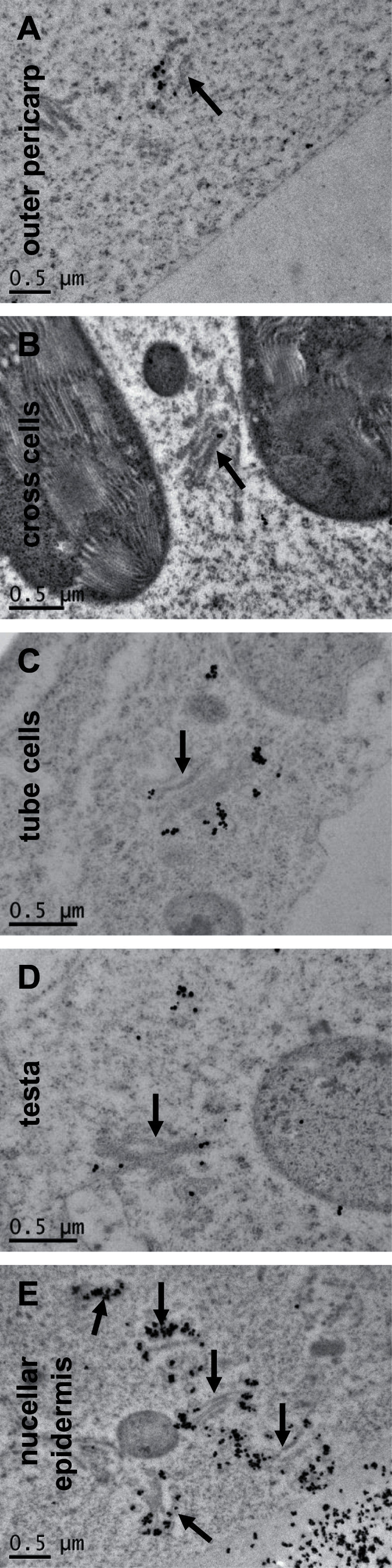
Golgi apparatus labelled with antibody specific to AX in the outer layers. Wheat grain sections labelled with anti-AX1 and observed with TEM. Micrographs of (A) outer pericarp, (B) cross cells, (C) tube cells, (D) testa and (E) nucellar epidermis. The arrows point at labelled Golgi apparatus.

Golgi stacks were observed in the different cell types of the intermediate layers and in the outer pericarp (Fig. 2). Immunolabelling carried out with an antibody specific to the hemicellulose arabinoxylan (AX) showed labelled Golgi apparatus in all outer cell layers (Fig. 2), therefore cell wall components are actively synthesized in the different tissues of the wheat grain at this stage.

### Microsomal fractions from three parts of the wheat grain at 250°D revealed different proportions of Golgi and ER

**Fig. 3.**
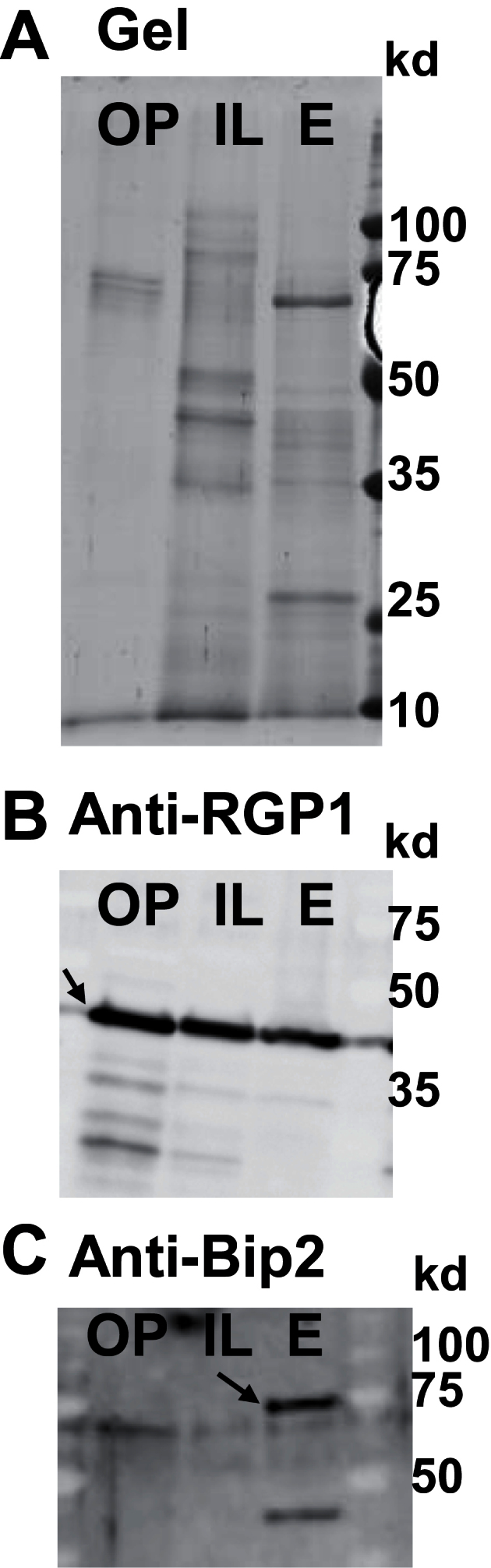
Characterization of microsomal fractions from outer pericarp, intermediate layers and endosperm. Proteins extracted from microsomal fractions were loaded on a SDS-PAGE gel and electroblotted on nitrocellulose membrane. The membrane was incubated with an antibody targeting the ER marker BiP2 and the Golgi marker RGP1. (A) SDS-PAGE stained with coomassie blue. (B) Immunoblot obtained with anti-RGP1. (C) Immunoblot obtained with anti-BiP2. A protein marker was loaded on gel to evaluate protein molecular weight. OP, outer pericarp; IL, intermediate layers; E, endosperm. The arrows point at the RGP the RGP1 and BiP2 bands.

The endomembrane content of the microsomal fractions was evaluated by western blot experiments using two antibodies: anti-RGP1, already described as labelling Golgi apparatus (Dhugga *et al.*, 1997; Suliman *et al.*, 2013), and anti-BiP2, a classical ER marker. The anti-RGP1 labelled microsomal fractions from endosperm, the reference fraction here, and also from intermediate layers and outer pericarp (Fig. 3B). The anti-BiP2 strongly labelled the microsomal fraction from the grain endosperm but the BiP2 band expected at 75kDa was not detected in the fractions from the outer pericarp and intermediate layers (Fig. 3C). Although less protein was extracted from the outer pericarp and loaded on the gel compared to the two other fractions, the ratio of Golgi/ER seemed higher in the microsomal fractions from outer pericarp and intermediate layers than in the one obtained for the grain endosperm (Fig. 3).

**Fig. 4.**
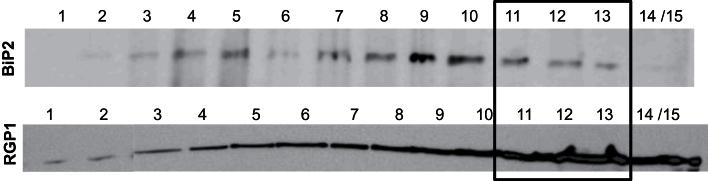
Western blot analysis of the 15 iodixanol fractions generated from the intermediate layers microsomes. Proteins extracted from microsomal fractions were loaded on a SDS-PAGE gel and electroblotted on nitrocellulose membrane. The membrane was incubated with anti-BiP2 (ER marker) anti-RGP1 (Golgi marker). Fractions 11–13 were selected.

In the case of the outer pericarp, the fractionation was stopped at this step because of the limited amount of tissue available even though ~ <1000 grains were dissected. A subcellular fractionation procedure was set up according to Suliman *et al.* (2013) to obtain fractions enriched in Golgi membranes from the intermediate layers. The corresponding microsomal fraction was further separated on an iodixanol gradient, and 15 fractions were collected and characterized. The western blots (Fig. 4) show the relative distribution of Golgi membranes and ER along the gradient fractions. The fractions 11–13, with a relative high content in RGP1 and, at the same time, a low content in BiP2, were selected.

Membranes were recovered from each of the selected fractions (one from the outer pericarp and three from the intermediate layer). In each case, the proteins were extracted, separated by electrophoresis, digested with trypsin and the resulting peptides were separated and analysed by nano LC MS/MS.

### Subcellular proteomics revealed cell wall-related protein families

While the sequencing of the hexaploid wheat genome is still underway, the genome of several related Poaceae species has been sequenced, annotated and published recently (Initiative IB, 2010; Brenchley *et al.*, 2012; Mayer *et al.*, 2012; Jia *et al.*, 2013; Ling *et al.*, 2013). To identify proteins, the UniProt database was restricted to Poacae and interrogated. First, a global analysis of all spectra generated from the pericarp microsomal fraction and from Golgi-enriched fractions from intermediate layers was performed. A total of 822 and 1304 proteins were identified respectively in the pericarp microsomal fraction and in the Golgi-enriched fractions from intermediate layers (Supplementary Table S2).

In order to identify all putative GTs, which were the primary targets, a BLAST search was run against the entire non-redundant sequences of the CAZy database. The interrogations resulted in many hits in CAZy in different families of GTs, GHs, proteins with carbohydrate binding modules (CBMs), carbohydrate esterases (CEs), proteins with auxilliary activities (AAs).

Several CAZy families have been implicated in cell wall metabolism and/or were localized in Golgi or in cell walls (Nikolovski *et al.*, 2012; Pellny *et al.*, 2012; Douché *et al.*, 2013). The CAZy families implicated in cell wall metabolism were manually examined. Additional cell wall-related proteins were identified in Supplementary Table S2 through their annotation. The annotation of all the gathered cell wall-related proteins were examined and completed if required with a tBLASTN analysis. Protein hits with confirmed relations with cell walls are reported in Supplementary Table S3 together with their proposed target polymer. A short list is given in Table 1.

**Table 1.** Short-list of cell wall-related proteins identified in outer pericarp (OP) and intermediate layers (IL)

Family	Uniprot ID	Annotation from Uniprot name or from tBlastN	species	polymer	OP	IL
GT2	W5DRH6	Cellulose synthase A	*T. aestivum*	cellulose	yes	yes
GT2	A6Q0E9	Mixed beta glucan synthase (CSLF6)	*T. aestivum*	MLG	yes	yes
GT2	A2YU42	Cellulose synthase-like protein (CSLD2)	*O. sativa*	mannan, cellulose	no	yes
GT43	Q50HV4	Xylan synthase (IRX14)	*T. aestivum*	xylan	no	yes
GT43	M5EF78	Xylan synthase (clade IRX9)	*T. aestivum*	xylan	no	yes
GT47	E0ZPV1	Beta-1,4-xylosyltransferase IRX10L-like	*T. aestivum*	xylan	yes	yes
GT48	M7ZA49	Callose synthase 9	*T. urartu*	callose	yes	yes
GT61	M8CXR2	Xylan arabinosyl transferase (xat1) clade A GT61	*A. tauschii*	AX	yes	yes
GT75	M8C7V8	Glycosyltransferase 75 (GT75-4)	*A. tauschii*	AX	yes	yes
GH3	M7ZGC8	Beta-D-xylosidase	*T. urartu*	xylan	yes	yes
GH3	W5F8K3	Beta-D-glucan exohydrolase	*T. aestivum*	MLG	yes	yes
GH3	W5ARE8	Alpha-L-arabinofuranosidase/beta-D-xylosidase isoenzyme ARA-I	*T. aestivum*	AX	yes	yes
GH9	M0VM74	Cellulase	*H. vulgare*	cellulose	no	yes
GH9	Q8RWR6	Endo-1,4-beta-glucanase	*T.aestivum*	cellulose	yes	no
GH16	Q41542	Xyloglucan endotransglucosylase/hydrolase	*T. aestivum*	xyloglucan	yes	no
GH17	M8BK19	Glucan endo-1,3-beta-glucosidase	*A. tauschii*	callose	yes	yes
GH28	R7VZ03	Polygalacturonase	*A. tauschii*	pectin	yes	yes
GH51	W5DRR0	Alpha-L-arabinofuranosidase 1	*T. aestivum*	AX	yes	yes
CE8	M8BPX2	Pectin methyl esterase inhibitor (domain PME and PMEi)	*A. tauschii*	pectin	yes	no
CE8	M8BV08	Pectin methyl esterase (domain PME only)	*A. tauschii*	pectin	no	yes
CE13	M7ZP69	Pectin pectin acetyl esterase	*T. urartu*	pectin	yes	yes
BAHD	A6Q0N9	AcylTase 4	*T. aestivum*	xylan, lignin, cutin	no	yes
COMT	M8BUF4	Caffeic acid 3-O-methyltransferase	*A. tauschii*	lignin	yes	yes
CCoAOMT	Q75RZ2	Caffeoyl CoA O-methyltransferase	*T. aestivum*	lignin	No	yes
CAD	M7Y7E2	Cinnamyl alcohol dehydrogenase	*T. urartu*	lignin	no	yes
GDSL	N1R0Q3	GDSL esterase/lipase	*A. tauschii*	cutin	yes	yes

### Correlations between polysaccharide distribution and identified proteins in the grain outer layers

The presence in the cell walls of the developing wheat grain at 250°D of several polysaccharides is documented in the literature (Beaugrand *et al.*, 2004; Philippe *et al.*, 2006; Pellny *et al.*, 2012; Chateigner-Boutin *et al.*, 2014). Table 2 presents a summary of published immunodetections with data available for the grain outer layers.

**Table 2.** Cell wall polysaccharide and hydroxycinnamic acid distribution in the different cell types of the wheat grain outer layers at 250°D

Probe	Target	Outer pericarp	Cross cells	Tube cells	Testa	Nucellus epidermis	References
INRA RU1	**RG1 backbone**	+	-	na	-	+	Chateigner-Boutin *et al.* (2014)
LM5	**(1–4) β galactan/**RGI side chain	-	-	na	-	+	Chateigner-Boutin *et al.* (2014)
LM6	**(1–5) β arabinan/** RGI side chain	-	-	na	-	+	Chateigner-Boutin *et al.* (2014)
LM19	**Low methylated HG**	-	-	na	+	-	Chateigner-Boutin *et al.* (2014)
LM20	**Methylated HG**	+	+	na	+	+	Chateigner-Boutin *et al.* (2014)
Anti-BG	**MLG**	+	+	+	+	+	Meikle *et al.* (1994); Philippe *et al.* (2006) here
CBM3a	**Cellulose**	+	+	+	+	+	Blake *et al.* (2006) Saulnier *et al.* (2012) Here
Anti-AX1	**AX**	+	+	+	+	+	Philippe *et al.* (2006) Here
UX1	**Glucuronoxylans**	+	-	-	+	-	Koutaniemi *et al.* (2012) Here
Anti-5-O- FerAra	**Fer AX**	+	+	+	+	+	Philippe *et al.* (2007) Here
INRA-COU1	**P-coumaric acid**	-	-	-	-	-	Tranquet *et al.* (2009) Here
LM15	**Xyloglucan**	+	+	+	+	-	Marcus *et al.* (2008) Here
LM21	**Mannan**	+	+	+	+	+	Marcus *et al.* (2010) Here
Anti-callose	**Callose**	+	+	+	+	+	Meikle *et al.* (1991) Here

na: non-available.

Immunolabellings were performed on sections of grains harvested at 250°D with several well established antibodies to complete the cell wall analysis. The aim was to detect the product/substrate of the different GT, GH and CE activities. The micrographs are shown in Supplementary Fig. S3.

CAZy families implicated in the synthesis of AX have been identified in this paper. The anti-AX1 antibody recognizing xylan substituted with arabinose (Guillon *et al.*, 2004) labelled all outer layers with a signal particularly strong in the wall of the nucellar epidermis. A similar pattern was observed with antibodies recognizing feruloylated AX but no signal was detected with an antibody targeting para-coumaric acid, another hydroxycinnamic acid reported to decorate xylan (Tranquet *et al.*, 2009). An antibody specific to glucuronoxylans revealed a weak labelling in the outer pericarp and a clear labelling only in the cell walls of the testa at 250°D. Interestingly only the cell walls separating the two cell layers of the testa were labelled, particularly in the cell corners.

GT48 proteins identified here have been implicated in the synthesis of callose. The anti-callose antibody revealed a signal in the cell walls of all outer cell layers at 250°D. The signal was mainly detected as patches of gold particles in the region of cell walls in contact with the plasma membranes.

GT2, and GH16 families have been implicated in the metabolism of mannan and xyloglucan. The antibody LM21 targeting mannan revealed a signal in the cell walls of all the outer grain tissues. The antibody LM15 targeting xyloglucan labelled the cell walls of the outer pericarp, cross cells, tube cells and testa but not the cell walls of the nucellar epidermis.

The GT2 protein CslF6 implicated in the synthesis of mixed-linked glucan (MLG) (Nemeth *et al.*, 2010) was identified in all fractions. The antibody specific to MLG strongly labelled the walls of all outer grain layers.

GT2 proteins of the CesA subgroup and GH9 identified here have been implicated in the metabolism of cellulose. To detect cellulose, the probe CBM3a is frequently used for dicot samples. In wheat grain, extra care has to be taken since CBM3a may cross-react with MLG. CBM3a labelling was detected in the walls of all grain outer layers. A lichenase treatment, which selectively degraded MLG and abolished MLG detection, did not prevent CBM3a labelling (Supplementary Fig. S4). The walls of the nucellar epidermis which were strongly labelled with anti-BG were only weakly labelled with CBM3a (Supplementary Fig. S3). Immunofluorescence assays and TEM results indicate that cellulose is present in the walls of all outer layers.

The results were incorporated into Table 2.

## Discussion

Several enzymes potentially involved in cell wall metabolism in the wheat grain endosperm were recently identified using a subcellular proteomics approach (Suliman *et al.*, 2013). A similar strategy was used here to identify enzymes responsible for cell wall metabolism in the grain outer layers. In parallel, information and new results were gathered concerning the polysaccharide composition of the cell walls in the different peripheral cell layers of the wheat grain using specific antibodies and probes. The developmental stage 250°D after flowering was chosen because the grain is then easily dissected and because there is an active deposition of cell wall polysaccharides.

Wheat grain fractions were prepared and enriched in Golgi membranes. The objective here was to simplify the content of the wheat grain fractions and enrich them in cell wall-related enzymes. The goal was neither to provide a comprehensive list of Golgi proteins nor to obtain a list of proteins with an absolute confidence in their Golgi location. GTs, GHs and CEs were identified in CAZy families known to contain cell wall-related enzymes and additional proteins which may constitute interesting candidates to investigate further.

Proteomic results obtained from the two grain fractions intermediate layers and pericarp are not directly comparable as the microsome fractionation step was not conducted for the outer pericarp. Moreover, not observing a protein using proteomics techniques does not mean it is absent in the sample. Dealing with protein redundancy is a difficult task for proteomic studies performed on species without a completely sequenced genome. As the Uniprot interrogations were carried out at the Poaceae level, several hits may correspond to the same wheat protein. Moreover the identification criteria of one single specific peptide may not be stringent enough, especially in protein families highly represented in databases.

Protein families implicated in cell wall metabolism were identified and several could be ascribed to cell wall components that are reported to be present in the cell walls of the different grain tissues.

Xylans are a class of polysaccharides that contain a backbone constituted of β(1–4) xylose residues. Xylans can be decorated with many other residues (Saulnier *et al.*, 2012). The precise distribution of the different structures of xylans in wheat cell layers is not fully established. Here specific antibodies targeting xylan substituted with either arabinose or (methyl)glucuronic acid revealed a differential distribution of the two forms of xylans. Several protein families known or proposed to be involved in xylan metabolism were identified here. GT43 and GT47 of the A subgroup (Zhong and Ye, 2003) have been implicated in the elongation of the xylan backbone (Brown *et al.*, 2007; Lovegrove *et al.*, 2013; Lee *et al.*, 2014). GT75, also known as RGPs, were shown to have a UDP-Ara*p* mutase (UAM) activity, which is required to convert the UDP-arabinopyranose into UDP-arabinofuranose, a precursor of AX (Konishi *et al.*, 2007). Several GT61 of the subgroup A, which was shown to contain xylan O3 arabinosyltransferases (XATs) (Anders *et al.*, 2012), were identified. Some of the arabinose residues linked to the xylan backbone are esterified with hydroxycinnamic acids, mainly ferulic acid but also para-coumaric acid. AcylCoA transferases of the BAHD family were retrieved in the list of proteins and AX feruloyltransferase were proposed to be member of this family, although clear experimental evidence is still lacking (Piston *et al.*, 2010; Molinari *et al.*, 2013). A coumaroyltransferase activity was attributed to a rice BAHD protein (Bartley *et al.*, 2013). Para-coumaric acid is proposed to mainly bind to lignins in grass cell walls but also to glucuronoarabinoxylans (Bartley *et al.*, 2013). Here an attempt to detect coumaric acid in wheat outer layer using the antibody INRA-COU1 failed. GHs of the GH3 and GH51 families that were detected in the grain outer layers were proposed to have xylan-related activities such as AX arabinofuranohydrolase (Ferré *et al.*, 2000, Lee *et al.*, 2001), arabinofuranosidase/xylosidase and xylosidase (Lee *et al.*, 2003).

MLG consist of linear chains of glucose residues linked by β(1–4) and β(1–3) linkages. This polymer, which can be abundant in grass cell walls, is not present in the cell walls of dicots. At the investigated developmental stage MLG have been found in cell walls of all grain outer layers. GT2 CslF6 protein was proposed to have MLG synthase activity since MLG-free *Arabidopsis* transformed with barley CSLF synthesized MLG (Burton *et al.*, 2006). GH3 were also implicated in the metabolism of MLG as MLG exohydrolase. GT2/CslF6 and GH3 MLG exohydrolase were identified in all grain tissues in accordance with the ubiquitous MLG detection (here and in Suliman *et al.*, 2013).

Cellulose is formed of glucose residues linked only by β(1–4) linkages. GT2/CesA and GH9 cellulases were identified in the pericarp and intermediate layers. Cellulose is present in the cell walls of all grain outer layers as revealed by labelling with CBM3a in this study. Cellulose was found to be abundant in the cell walls of the outer pericarp and intermediate layers by Barron *et al.* (2007).

Callose is another glucan in which glucose residues are linked only by β(1–3) linkages. Callose synthase activity was ascribed to members of the GT48 family (Verma and Hong, 2001). In this work, GT48 proteins were detected in all grain outer tissues. Deposition of callose was observed in the wheat endosperm during grain development (Philippe *et al.*, 2006; Pellny *et al.*, 2012). Here callose was detected at 250°D in the cell wall of all outer layers. The GH17 family includes β-1,3-glucanases that specifically degrade callose (Levy *et al.*, 2007). Several GH17s were identified in this experiment that may be involved in the degradation of callose.

Mannans are polysaccharides known to be present in the cell wall of wheat endosperm (Mares and Stone, 1973) and mannans were detected here in the cell wall of all grain outer tissues at 250°D. Virtually nothing is known about the structure and function of mannan in wheat. GT2 of the CSLA and CSLD subgroups have been implicated in the synthesis of mannan (Verhertbruggen *et al.*, 2011). Wheat CSLD2 was identified in the endosperm and intermediate layers (here and in Suliman *et al.*, 2013).

Xyloglucans are polysaccharides consisting of a chain of β(1–4) glucan decorated with xylose and several other types of residues depending on the species investigated. Xyloglucans were detected in wheat bran fractions and in wheat grain endosperm (Dupont and Selvendran, 1987; Pellny *et al.*, 2012). Here xyloglucans were found at 250°D in the cell walls of the pericarp and some (cross cells and tube cells) but not all cell layers included in the intermediate layers. A GH16 annotated xyloglucan endotransglycosylase/hydrolase (Q41542 XTH) was identified in the outer pericarp.

Recently the discovery of pectin in the cell walls of wheat grain was reported (Chateigner-Boutin *et al.*, 2014). Pectin is a complex polysaccharide composed of different domains that are covalently linked together (Mohnen, 2008). At 250°D, several of these domains were detected in the grain. Rhamnogalacturonan I (RGI) was found in the outer pericarp and the nucellar epidermis. Homogalacturonans (HGs) were revealed in the outer pericarp and intermediate layers (Table 2). The current model states that HGs are synthesized in the Golgi apparatus in a highly methylated form and subsequently demethylated by PMEs (Carpita and Gibeaut, 1993; Wolf *et al.*, 2009). At the 250°D stage, methylated HGs were detected in the grain outer layers and non (or low) methylated HGs only in the testa (Chateigner-Boutin *et al.*, 2014). In this survey, several proteins, the homologues of which have been implicated in pectin metabolism in other species, were identified. A CE8 protein annotated PME was identified in the intermediate layer and could correspond to the activity that removes the homogalacturonan methyl esters in the testa. The other CE8 identified possess both an esterase domain and a PME inhibitor domain which characterizes PME inhibitors (PMEi) (Wang *et al.*, 2013). Their action would prevent the removal of methyl groups from homogalacturonans. Members of the GH28 family containing the pectin-degrading polygalacturonase activity were detected in all grain fractions. CE13 identified in the outer layers were annotated pectin acetylesterase, this activity would remove acetyl groups from wheat pectin, which would suppose that pectin in wheat grain is acetylated as is the case in other species (Gou *et al.*, 2012).

This proteomic study revealed proteins acting on cell wall polysaccharides and also proteins involved in the synthesis of other cell wall components such as lignins and cutin. Caffeic acid *O*-methyltransferase (COMT), caffeoyl-CoA *O*-methyltransferase (CCoAOMT), cinnamyl alcohol deshydrogenase (CAD) proteins and AA proteins of family 1 and 2, which contain lignin peroxidase and laccase activities, were identified in the grain outer layers. COMT, CAD and CCoAOMT are involved in the synthesis of monolignols, the subunits of which form lignins upon polymerization in cell walls induced by the action of peroxidase and laccase (Vanholme *et al.*, 2010; Wang *et al.*, 2013). Lignins were found in the wheat grain outer layers (Schwarz et al., 1988; Antoine *et al.*, 2003). Several GDSL esterase/lipases were identified in the grain outer layers. GDSL esterase/lipases were implicated in the formation of the cuticle (Girard *et al.*, 2012), a complex structure containing waxes and cutin. Cell wall polysaccharides are associated with components of the cuticle and the cuticle has been proposed to be considered as a cutinized cell wall (Dominguez et al., 2011; Shi *et al.*, 2011; Guzmán *et al.*, 2014). In the wheat exocarp, testa and nucellar epidermis a cuticle is formed during grain development on the outside of the cell wall (Morrison, 1975). The GDSL esterase/lipases identified here may be implicated in the synthesis of the grain cuticles.

Many putative enzymes acting on cell walls were identified in this study. Differences were observed in the protein sets detected in the pericarp and in the intermediate layers. Some proteins were identified in all grain tissues; this is the case for instance for the GT47/E0ZPV1 involved in xylan synthesis. Others, such as GT43s in the intermediate layers and GH16/Q41542 in the outer pericarp were identified in only one tissue fraction. In some cases different members of the same family of proteins were identified in the pericarp and intermediate layers, therefore for the same activity different individual proteins might be represented in the different grain tissues.

The variability of cell wall composition between the different layers of the grain revealed by immunodetection is striking. The physiological significance of such a variability is far from understood. Insights may be brought by functional analysis on wheat altered in specific components of cell wall synthesis or modification machinery. Further investigation of the biochemical mechanism controlling the composition of cell walls could provide useful information to improve the quality (e.g. dietary fibre content) of cereal based products.

## Supplementary data

Supplementary Fig. S1. Experimental scheme with the steps followed to identify CAZy proteins in wheat grain outer pericarp and intermediate layers.

Supplementary Fig. S2. Brightfield micrographs of the dissected outer pericarp, intermediate layers and endosperm.

Supplementary Fig. S3. Transmission electron micrographs showing polysaccharides detected in cell walls of wheat grain outer layers at 250°D. Wheat grain sections were incubated with specific primary antibodies and secondary antibodies coupled with gold particles.

Supplementary Fig. S4. Immunofluorescence imaging of wheat grain sections labelled with anti-BG without and with prior removal of MLG by lichenase treatment, and with CBM3a after lichenase treatment.

Supplementary Table S1. Antibodies and probe used for cell wall analyses.

Supplementary Table S2. List of proteins identified in wheat grain outer pericarp and intermediate layers.

Supplementary Table S3. List of cell wall proteins identified in wheat grain outer pericarp and intermediate layers.

Supplementary Data

## Abbreviations:

AA: auxilliary activitiy
AX: arabinoxylan
BiP: binding immunoglobulin protein
CAZy: carbohydrate active enzymes
CBM: carbohydrate-binding module
CE: carbohydrate esterase
GH: glycosylhydrolase
GT: glycosyltransferase
LC MS/MS: liquid chromatography-tandem mass spectrometry
MLG: (1–3) (1–4) β-d-glucan or mixed-linked glucan
RGP: reversibly glycosylated protein.
