# Supplementary material for: Endomembrane proteomics reveals putative enzymes involved in cell wall metabolism in wheat grain outer layers
Source: J Exp Bot. 2015 Mar 13;66(9):2649–58. doi: 10.1093/jxb/erv075 (PMC4986875; doi:10.1093/jxb/erv075)
Supplement: Supplementary Data [file supp_66_9_2649__index.html]

Endomembrane proteomics reveals putative enzymes involved in cell wall metabolism in wheat grain outer layers — Endomembrane proteomics reveals putative enzymes involved in cell wall metabolism in wheat grain outer layers — Supplementary Data 

# Endomembrane proteomics reveals putative enzymes involved in cell wall metabolism in wheat grain outer layers

## Supplementary Data

Data files

**Files in this Data Supplement:**

- Supplementary Data - Supplementary Data
- Supplementary Data - Supplementary Data
