# Supplementary material for: Endomembrane proteomics reveals putative enzymes involved in cell wall metabolism in wheat grain outer layers
Source: J Exp Bot. 2015 Mar 13;66(9):2649–58. doi: 10.1093/jxb/erv075 (PMC4986875; doi:10.1093/jxb/erv075)

Endomembrane proteomics revealed putative enzymes involved in cell wall metabolism in wheat grain outer layers

Anne-Laure Chateigner-Boutin, Muhtadi Suliman, Brigitte Bouchet, Camille Alvarado, Hélène Rogniaux, Fabienne Guillon, Colette Larré

Supplementary table S1 : Antibodies and probes used for cell wall analyses.

Name	Source/ 2ary Ab	Specificity	Dilution	References
Anti-BiP2	rabbit	Anti-BiP2 from Arabidopsis	1:5000	Agrisera AS09 481
Anti-RGP1	rabbit	Anti-RGP1 from pea	1:10 000	Duggha et *al.*, 1997
Anti-callose	mouse	Linear beta 1-3 glucan oligosaccharide	1:100	Meikle et al 1991 Biosupplies Australia
UX1	mouse	Glucopyranosyl uronic acid or its 4-O-methyl ether substituents in xylan	1:3	Koutaniemi et al. 2012
CBM3a	Anti-histidine (1:100) mouse	CBM3a is a histidine tagged recombinant CBM from *Clostridium thermocellum* directed to crystalline cellulose .	1:1000	Blake et al 2006 PlantProbes
LM15	rat	Generated against a xylosylated heptasaccharide from tamarind xyloglucan (XXXG-BSA). Recognises the XXXG motif and can accommodate a single galactosyl residue.	1:5	Marcus et al 2008 PlantProbes
LM21	rat	LM21 binds to β-(1→4)-manno-oligosaccharides from DP2 to DP5 and displays a wide recognition of mannan, glucomannan and galactomannan.	1:5	Marcus et al 2010
Anti-AX1	mouse	Low substituted arabinoxylans	1:20	Guillon et al. 2004
Anti-5-O-Fer Ara	rabbit	5-O-trans-feruloyl L arabinose	1:5000	Philippe et al. 2007
Anti-BG	mouse	Linear (1-3) (1-4) β-D-oligosaccharide	1 :200	Meikle et al 1994 Biosupplies Australia
INRA-COU1	mouse	Free p-coumaric acid or esterified to arabinose	1:3	Tranquet et al. 2009

References

Dhugga KS, Tiwari SC, Ray PM. 1997. A reversibly glycosylated polypeptide (RGP1) possibly involved in plant cell wall synthesis: purification, gene cloning, and trans-Golgi localization. Proc Natl Acad Sci U S A 94, 7679-7684.

Meikle PJ, Bonig I, Hoogenraad NJ, Clarke AE, Stone BA. 1991. The location of (1→3)-β-glucans in the walls of pollen tubes of Nicotiana alata using a (1→3)-β-glucan-specific monoclonal antibody. Planta 185, 1-8.

Koutaniemi S, Guillon F, Tranquet O, Bouchet B, Tuomainen P, Virkki L, Petersen HL, Willats WG, Saulnier L, Tenkanen M. 2012. Substituent-specific antibody against glucuronoxylan reveals close association of glucuronic acid and acetyl substituents and distinct labeling patterns in tree species. Planta 236,739-51.

Blake AW, McCartney L, Flint JE, Bolam DN, Boraston AB, Gilbert HJ, Knox JP. 2006. Understanding the Biological Rationale for the Diversity of Cellulose-directed Carbohydrate-binding Modules in Prokaryotic Enzymes. The Journal of Biological Chemistry 281, 29321-29329.

Marcus SE, Verhertbruggen Y, Hervé C, Ordaz-Ortiz JJ, Farkas V, Pedersen HL, Willats WG, Knox JP. 2008. Pectic homogalacturonan masks abundant sets of xyloglucan epitopes in plant cell walls. BMC Plant Biol 8, 60 doi:10.1186/1471-2229-8-60.

Marcus SE, Blake AW, Benians TAS et al. 2010. Restricted access of proteins to mannan polysaccharides in intact plant cell walls. Plant J 64, 191–203.

Guillon F, Tranquet O, Quillien L, Utille J-P, Ordaz Ortiz JJ, Saulnier L. 2004. Generation of polyclonal and monoclonal antibodies against arabinoxylans and their use for immunocytochemical location of arabinoxylans in cell walls of endosperm of wheat. Journal of Cereal Science 40, 167-182

Philippe S, Tranquet O, Utille JP, Saulnier L, Guillon F. 2007. Investigation of ferulate deposition in endosperm cell walls of mature and developing wheat grains by using a polyclonal antibody. Planta 225, 1287-1299.

Meikle PJ, Hoogenraad NJ, Bonig I, Clarke AE, Stone BA. 1994. A (1-->3,1-->4)-beta-glucan-specific monoclonal antibody and its use in the quantitation and immunocytochemical location of (1-->3,1-->4)-beta-glucans. Plant J. 5,1-9.

Tranquet O, Saulnier L, Utille JP, Ralph J, Guillon F. 2009. Monoclonal antibodies to p-coumarate. Phytochemistry 70, 1366-1373.

FigS1 : Experimental scheme with the steps followed to identify CAZY proteins in wheat grain outer pericarp and intermediate layers.

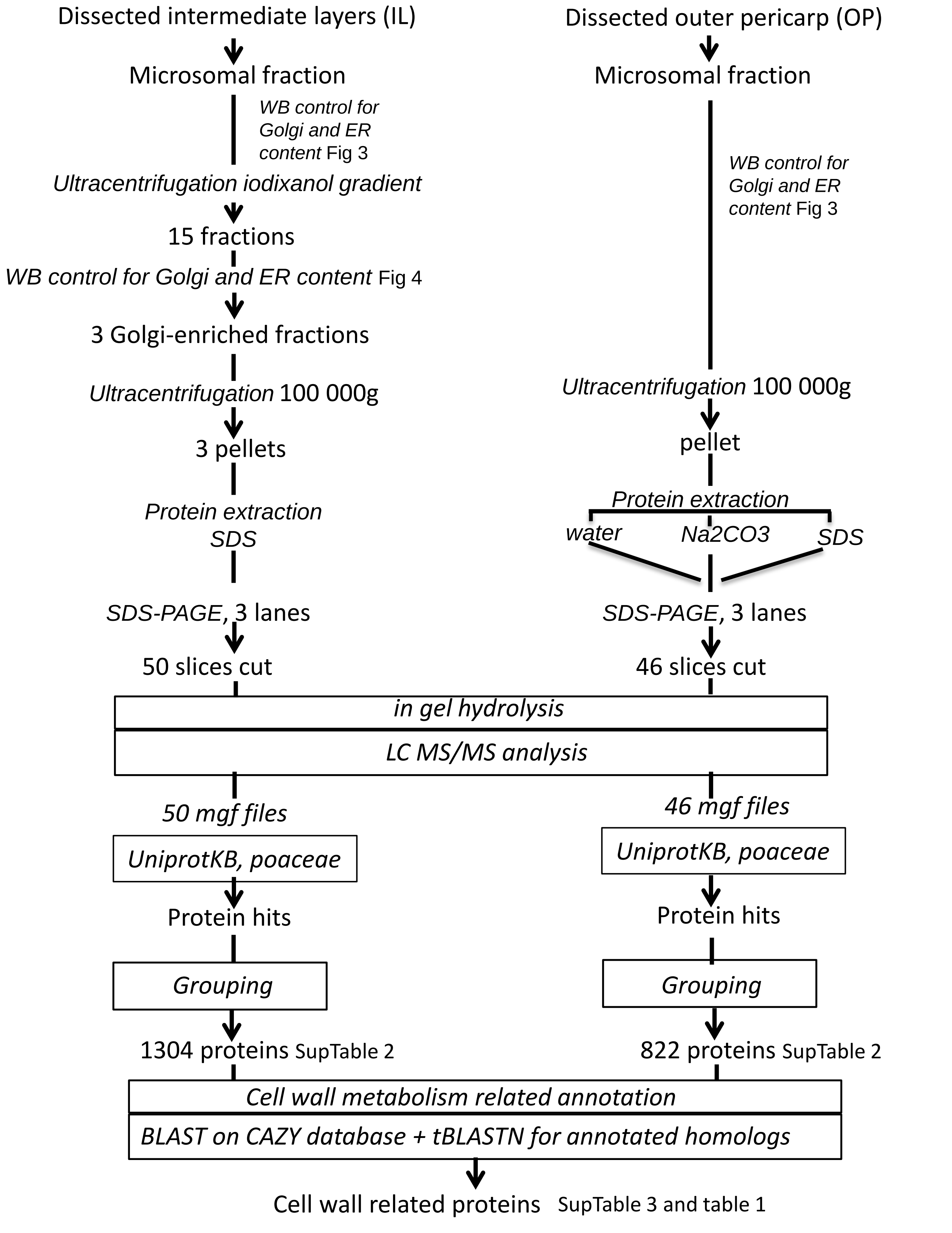

WB : western blot

Fig.S2. Brightfield micrographs of the dissected outer pericarp, intermediate layers and endosperm.

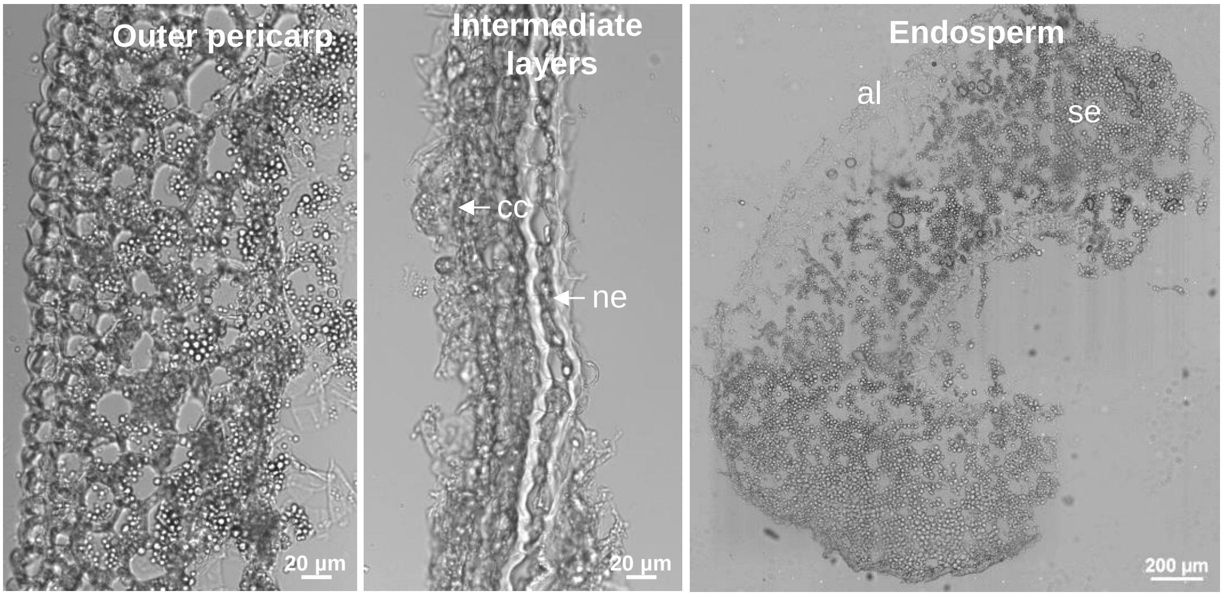

Cc: cross cells; ne: nucellar epidermis; al: aleurone; se: starchy endosperm .

Fig S3: Transmission electron micrographs showing polysaccharides detected in cell walls of wheat grain outer layers at 250°D. Wheat grain sections were incubated with specific primary antibodies and secondary antibodies coupled with gold particles.

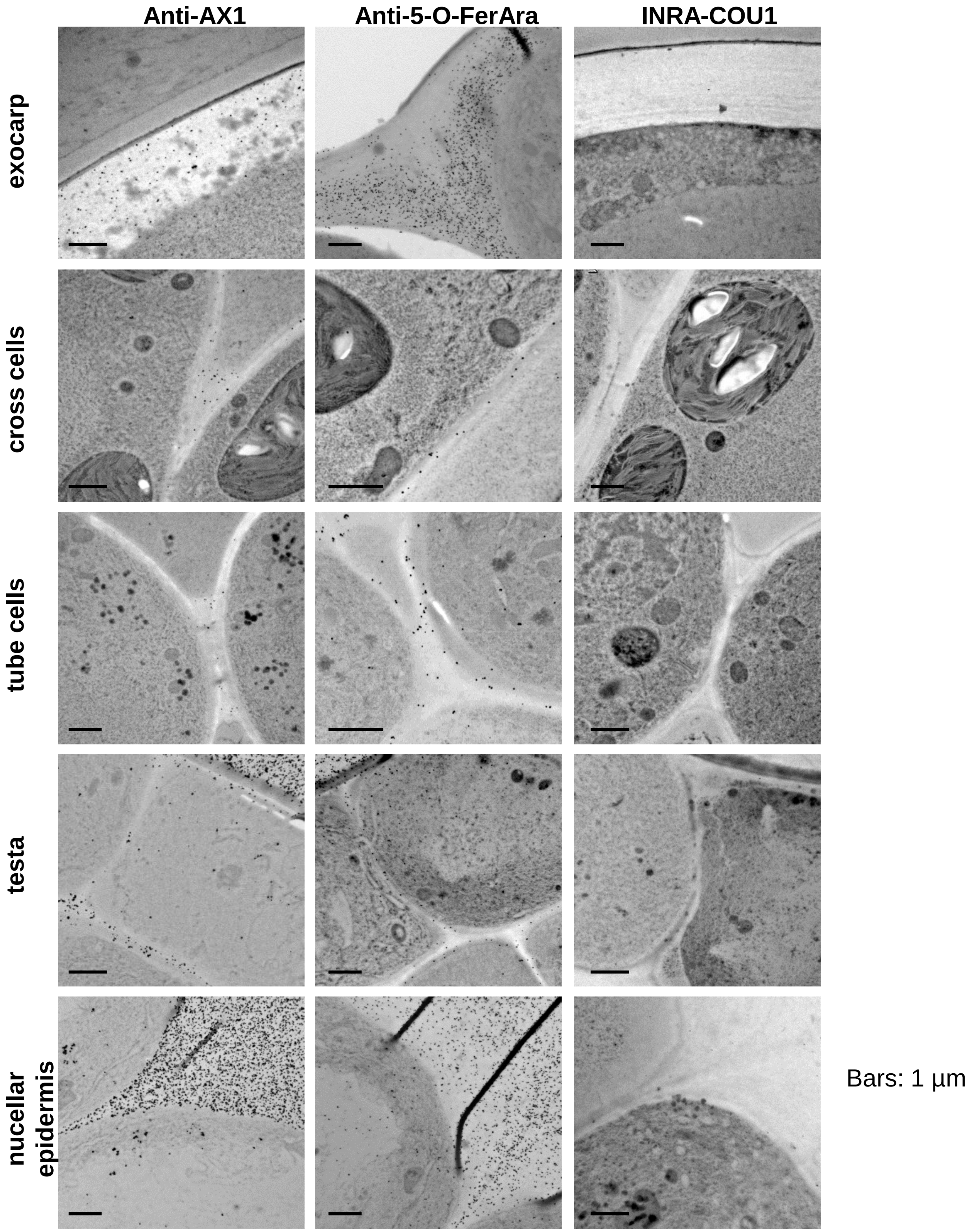

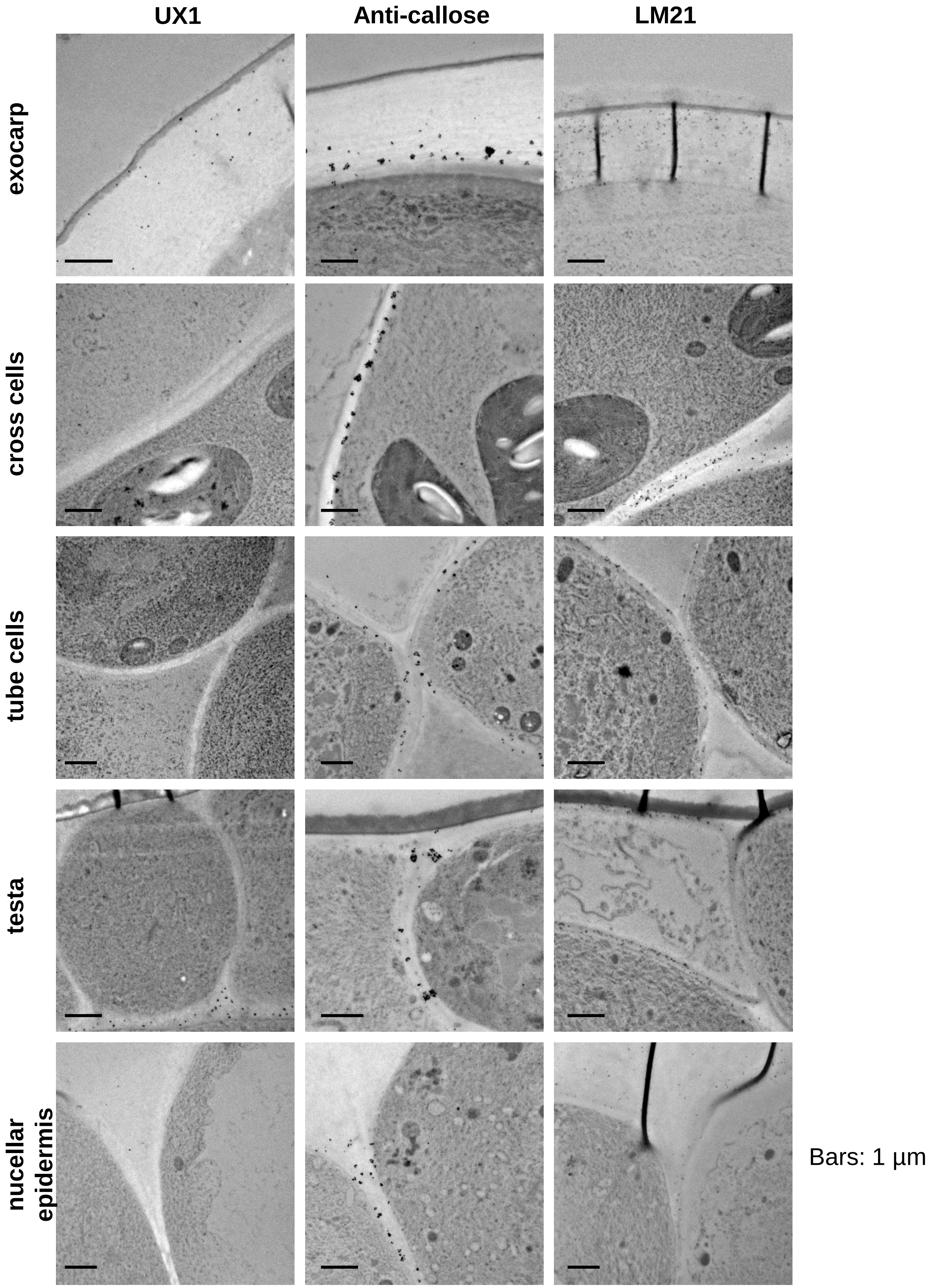
UX1
Anti-callose
LM21
exocarp
cross cells
tube cells
testa
nucellar epidermis
Bars: 1 µm

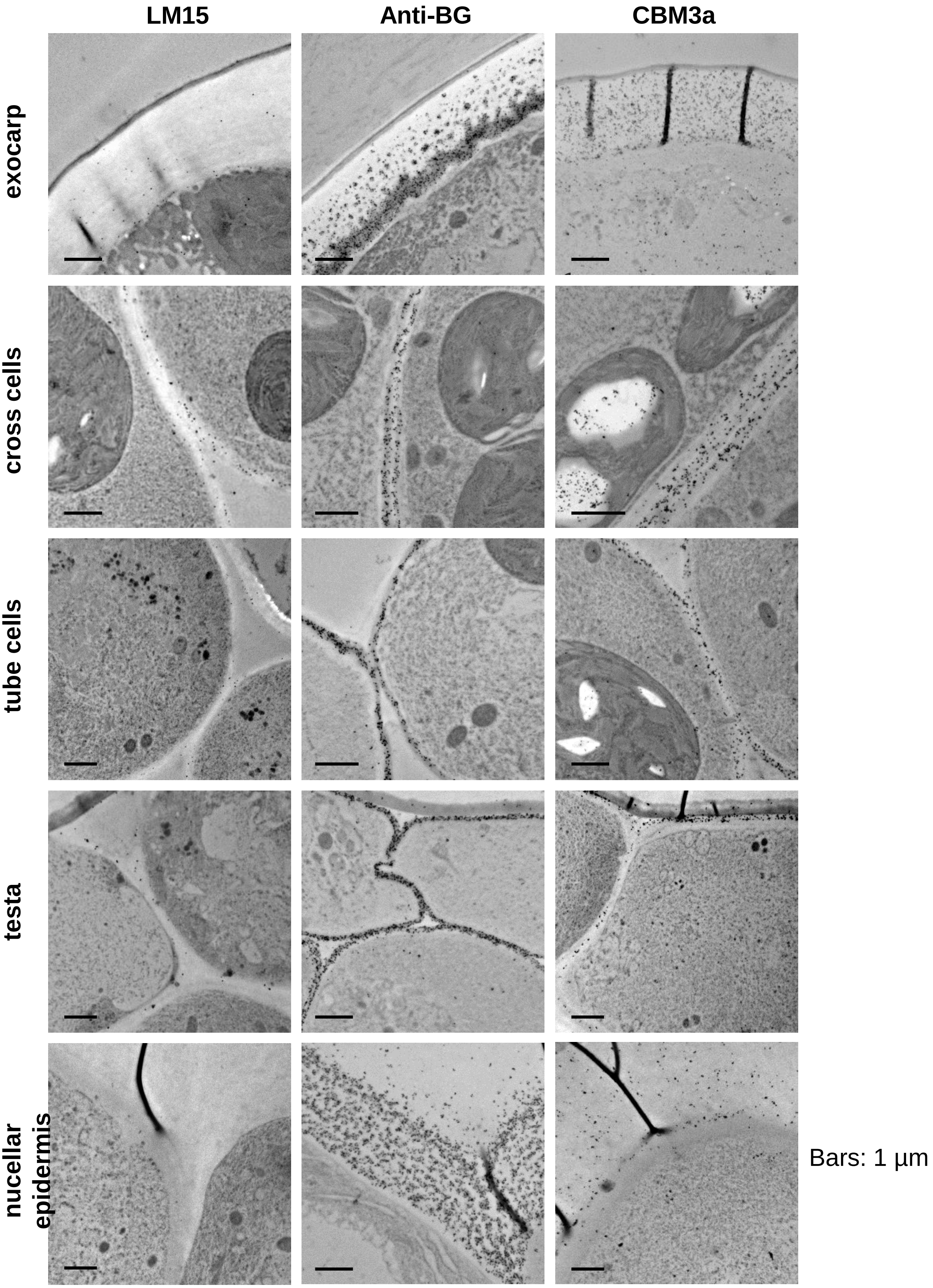
LM15
Anti-BG
CBM3a
exocarp
cross cells
tube cells
testa
nucellar epidermis
Bars: 1 µm

Fig.S4. Immunofluorescence imaging of wheat grain sections labeled with anti-BG without and with prior removal of MLG by lichenase treatment, and with CBM3a after lichenase treatment.

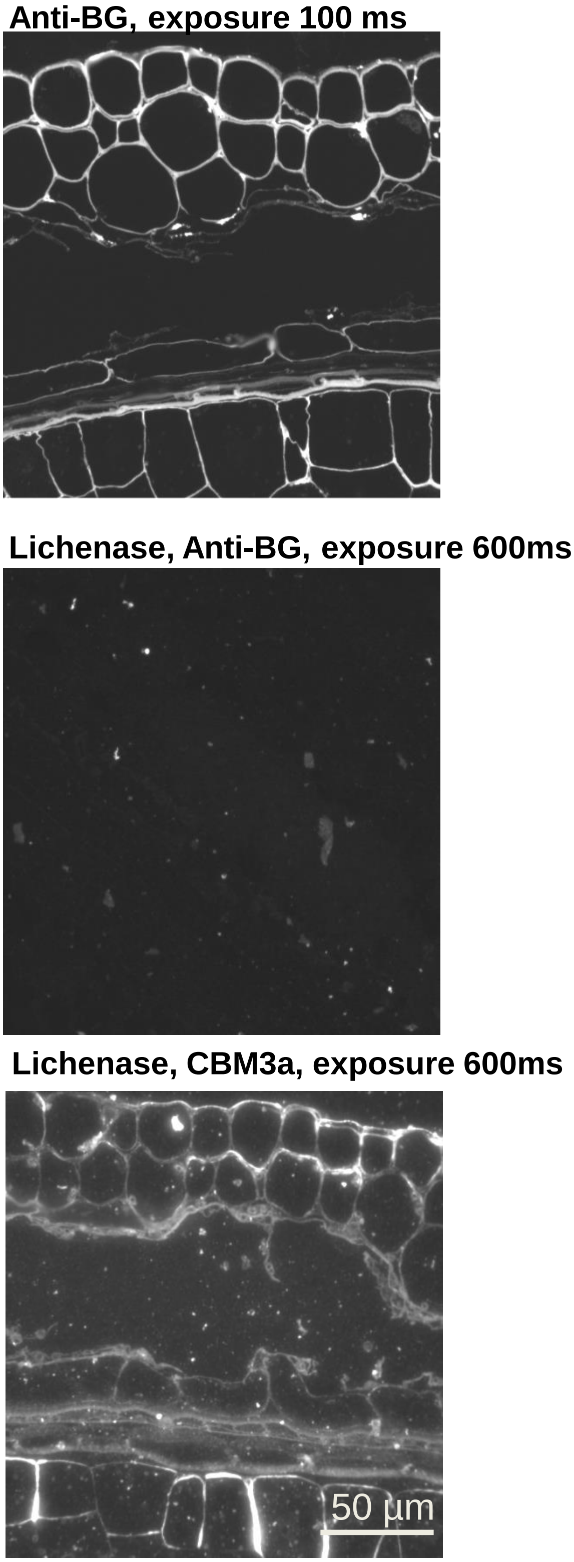

Supplement: Supplementary Data [file supp_erv075_jexbot129494_file002.pdf]
